# The role of the dopamine D1 receptor in social cognition: studies using a novel genetic rat model­

**DOI:** 10.1242/dmm.024752

**Published:** 2016-10-01

**Authors:** Judith R. Homberg, Jocelien D. A. Olivier, Marie VandenBroeke, Jiun Youn, Arabella K. Ellenbroek, Peter Karel, Ling Shan, Ruben van Boxtel, Sharon Ooms, Monique Balemans, Jacqueline Langedijk, Mareike Muller, Gert Vriend, Alexander R. Cools, Edwin Cuppen, Bart A. Ellenbroek

**Affiliations:** 1 Donders Institute for Brain, Cognition and Behaviour, Department of Cognitive Neuroscience, Radboud University Medical Centre, Nijmegen 6525 EZ, The Netherlands; 2 Department of Neurobiology, Unit Behavioural Neuroscience, Groningen Institute for Evolutionary Life Sciences, University of Groningen, Groningen 9700 CC, The Netherlands; 3 Victoria University of Wellington, School of Psychology, PO Box 600, Wellington 6040, New Zealand; 4 Hubrecht Institute, KNAW and University Medical Centre Utrecht, Utrecht 3584 CT, The Netherlands; 5 CMBI, Radboud University Nijmegen Medical Centre, Geert Grooteplein 26–28, Nijmegen 6525 GA, The Netherlands

**Keywords:** Dopamine D1 receptor, Mutant rat, Social cognition, Characterization, Schizophrenia

## Abstract

Social cognition is an endophenotype that is impaired in schizophrenia and several other (comorbid) psychiatric disorders. One of the modulators of social cognition is dopamine, but its role is not clear. The effects of dopamine are mediated through dopamine receptors, including the dopamine D1 receptor (Drd1). Because current Drd1 receptor agonists are not Drd1 selective, pharmacological tools are not sufficient to delineate the role of the Drd1. Here, we describe a novel rat model with a genetic mutation in Drd1 in which we measured basic behavioural phenotypes and social cognition. The I116S mutation was predicted to render the receptor less stable. In line with this computational prediction, this Drd1 mutation led to a decreased transmembrane insertion of Drd1, whereas *Drd1* expression, as measured by *Drd1* mRNA levels, remained unaffected. Owing to decreased transmembrane Drd1 insertion, the mutant rats displayed normal basic motoric and neurological parameters, as well as locomotor activity and anxiety-like behaviour. However, measures of social cognition like social interaction, scent marking, pup ultrasonic vocalizations and sociability, were strongly reduced in the mutant rats. This profile of the Drd1 mutant rat offers the field of neuroscience a novel genetic rat model to study a series of psychiatric disorders including schizophrenia, autism, depression, bipolar disorder and drug addiction.

## Abstract (precis)

**Summary:** Rats with a novel dopamine D1 receptor mutation, which causes reduced membrane Drd1 expression, display impaired social cognition, although their exploratory and anxiety-like behaviours are unaffected.

## INTRODUCTION

It is now commonly agreed that psychiatric disorders are not fully independent syndromes, but rather represent constellations of symptom clusters that partially overlap. In support of this idea, there is extensive comorbidity between psychiatric disorders. For instance, patients with schizophrenia often show symptoms of major depressive disorder, autism spectrum disorders, bipolar disorders and/or drug addiction. Intriguingly, a very recent report analysing structural magnetic resonance imaging (MRI) studies has reported that schizophrenia, and several major psychiatric disorders are jointly characterized by changes in brain areas mediating social cognition (Goodkind et al., 2015). The ‘social brain’ is indeed a common factor in schizophrenia and its comorbid disorders (Fett et al., 2015; Hoertnagl and Hofer, 2014; Pinkham, 2014). Social cognition can be defined as all processes that are elicited by and/or directed towards other subjects (Kennedy and Adolphs, 2012). Increasing effort has been made to understand the intricacies of the social brain (Kennedy and Adolphs, 2012; Meyer-Lindenberg and Tost, 2012). One of the major players is dopamine.

Research in non-human primates and rodents has shown that amphetamine, a dopamine-releasing drug, reduces affiliative social behaviour (Ellenbroek et al., 1989; Schiorring, 1979). However, it has also been reported that pro-social behaviour (induced by playing back 50 kHz ultrasonic vocalizations that elicit approach behaviour) is associated with increased dopamine release (Willuhn et al., 2014). These conflicting data might in part be related to opposing roles of different dopamine receptors. In this study, we focus on the role of the dopamine D1 receptor (Drd1). A recent positron emission tomography (PET) scan study in healthy volunteers showed that Drd1 binding was positively correlated to social conformity (Plaven-Sigray et al., 2014). In line with this, studies in zebrafish have found that Drd1 antagonists reduced social preference (Scerbina et al., 2012). By contrast, studies in male macaque monkeys have shown that a Drd1 antagonist actually improved social behaviour that was disrupted by amphetamine (Ellenbroek et al., 1989). Likewise, local injections of a Drd1 antagonist into the nucleus accumbens of mice leads to increases in social approach behaviour in females but not males (Campi et al., 2014). Finally, Drd1 agonists injected into the nucleus accumbens of male prairie voles prevent new pair bonding, but facilitated the maintenance of pair bonding when the agonist was administered after a bond was formed (Aragona et al., 2006).

Taken together, these data emphasize that, although Drd1 clearly plays an important role in social cognition, its precise function is far from understood. The conflicting data might – at least in part – be due to differences in the behavioural paradigm being used, as well as differences in species and sex. Perhaps most importantly, most studies investigating the role of Drd1 in social cognition have relied on pharmacological manipulations of Drd1. Unfortunately, none of the drugs acting on Drd1 show selectivity for Drd1 over other dopamine receptors, and several of these drugs also show affinity for serotonin receptors (Undieh, 2010). Therefore, in order to investigate the role of Drd1 in social cognition as a transdiagnostic marker for schizophrenia and comorbid psychiatric disorders, we introduce in the present paper a novel rat model with a mutation in the *Drd1* gene. We first provide evidence that the mutant Drd1 is dysfunctional. Subsequently, we measured basic behaviour and social cognition in the rat carrying the Drd1 mutation. To assess social cognition we used social interaction, social approach and avoidance, olfactory-scent marking and social anxiety (maternal-separation-induced ultrasonic vocalizations) tests. Our data show that the receptor Drd1 is crucially involved in all these aspects of social cognition. As these social traits are also prominent in schizophrenia and comorbid disorders, we believe our rat model to be useful for research into these disease states.

## RESULTS

We treated male Wistar rats with *N*-ethyl-*N*-nitrosourea (ENU) to induce mutagenesis (Smits et al., 2006) and identified a rat among the offspring with a missense mutation in the gene encoding for Drd1. The mutation involves a hydrophobic isoleucine into polar serine residue exchange (Drd1^I116S^) in helix III at position 116 of the protein. We outcrossed this mutant rat for at least five generations to wild-type Wistar rats to generate a Drd1 mutant rat model. We characterized and tested this mutant rat model for basic behavioural phenotypes and social cognition.

### Characterization of the Drd1^I116S^ mutant rat

#### Computational modelling of the Drd1^I116S^ receptor

**Fig. 1.**
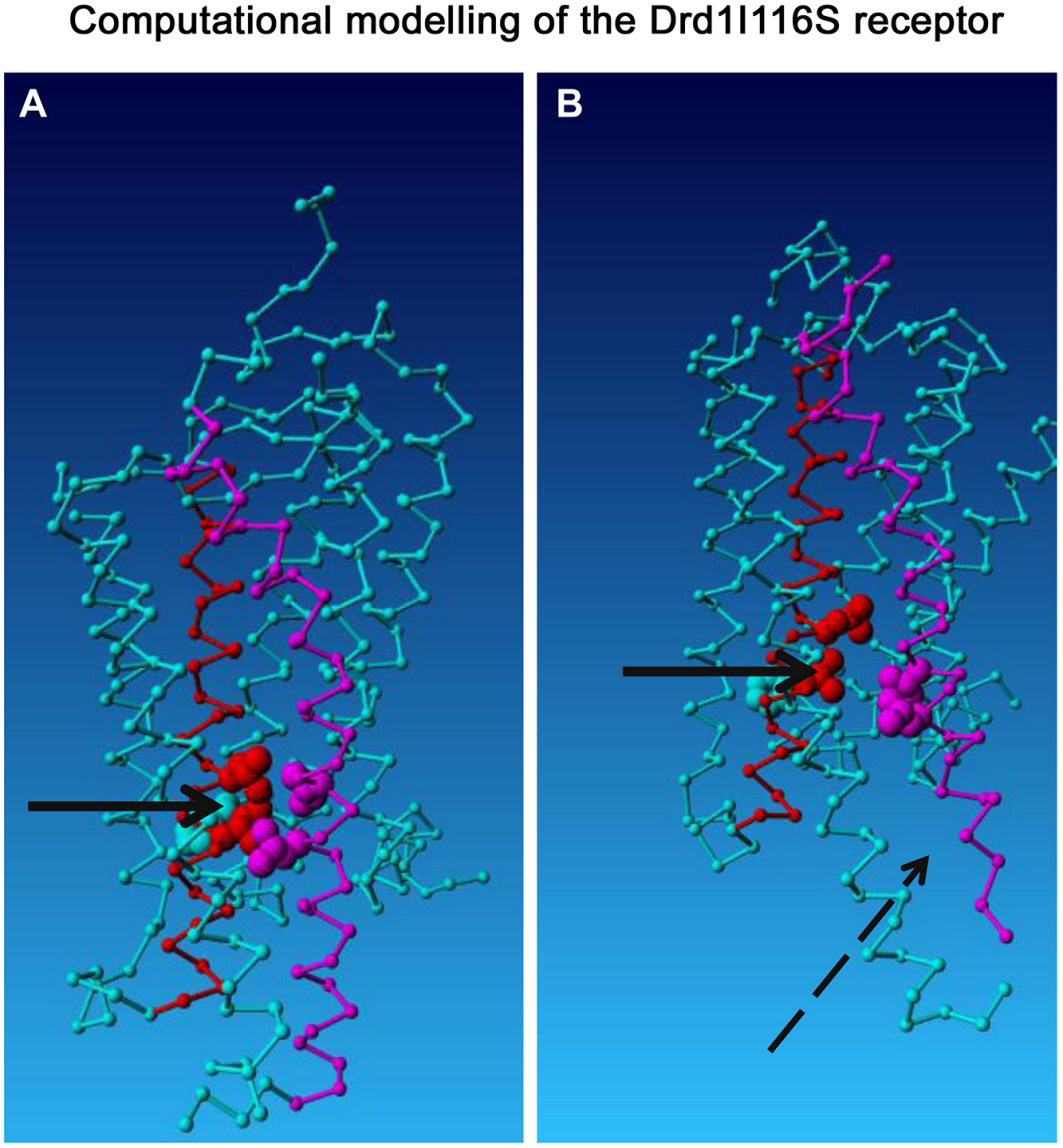
**Computational modelling of the wild-type Drd1 and the Drd1^I116S^ protein structure.** A computational model of (A) the wild-type Drd1 receptor, and the (B) Drd1^I116S^ receptor is shown. The solid arrow indicates the position of the I116S mutation. The purple amino acids reflect helix VI, and the red amino acids reflect helix III. Ligand binding takes places at about two to three helical turns above the solid arrow. The I116S mutation (located just inside the membrane near the cytosolic side of helix III) breaks a series of hydrophobic contacts with amino acids 276 and 277 (valine and isoleucine) in helix VI and with the highly conserved R120 at the extracellular side of helix III. The exact position of this arginine residue, and the contacts between helices III and VI are important for maintaining the receptor in an inactive state. The I116S mutant disturbs the position of R120 and the interactions between helices III and VI. This might lead to a repulsion of helix VI (broken arrow), an opening for G-protein binding (lower side of the displayed protein) and activation and instability of the receptor.

We first performed a computational analysis and modelling of the Drd1^I116S^ mutation. The ENU-induced mutation changed a hydrophobic isoleucine residue into a hydrophilic serine residue at position 116. The Drd1 protein belongs to the D1-like family of dopamine receptors that couples to the intracellular G protein G-stimulatory (Gs). As illustrated in Fig. 1, computational modelling predicted that, in Drd1^I116S^, the cytoplasmic ends of helix III and helix VI cannot approach each other as closely as in the wild-type Drd1, breaking contact with the highly conserved R120 at the extracellular side of helix III that binds to G proteins. The separation of the cytoplasmic ends of helix III and helix VI might open this part of the receptor to a larger extent, meaning that the receptor could be rendered instable.

#### Dopamine receptor mRNA levels

Next, to assess whether the Drd1^I116S^ mutation affects *Drd1* transcript levels, we determined mRNA expression of *Drd1* in the striatum (rich in dopamine) by quantitative PCR (qPCR). We also assessed mRNA levels of other dopamine receptor subtypes (*Drd2*–*Drd5*), to evaluate whether a change in Drd1 would indirectly affect the expression of genes encoding for other dopamine receptors. We found that the mRNA levels of *Drd1* (Z=−1.389, *P*=0.165), *Drd2* (Z=−0.579, *P*=0.563), *Drd3* (Z=−0.000, *P*=1.000), *Drd4* (Z=−0.347, *P*=0.728) and *Drd5* (Z=0.000, *P*=1.000) were unaltered in the striatum of wild-type rats compared to Drd1^I116S^ mutant rats (Fig. S1). *Drd4* mRNA levels were very low in both wild-type and mutant rats, which corresponds to previous findings for *Drd4* mRNA levels in the striatum of wild-type subjects (Matsumoto et al., 1996). None of the dopamine receptors showed any difference in levels between the wild-type and Drd1 mutant genotypes, suggesting that the Drd1^I116S^ mutation does not affect Drd1 transcript stability and/or degradation, nor that of the other dopamine receptors.

### Drd1^I116S^ receptor binding

**Fig. 2.**
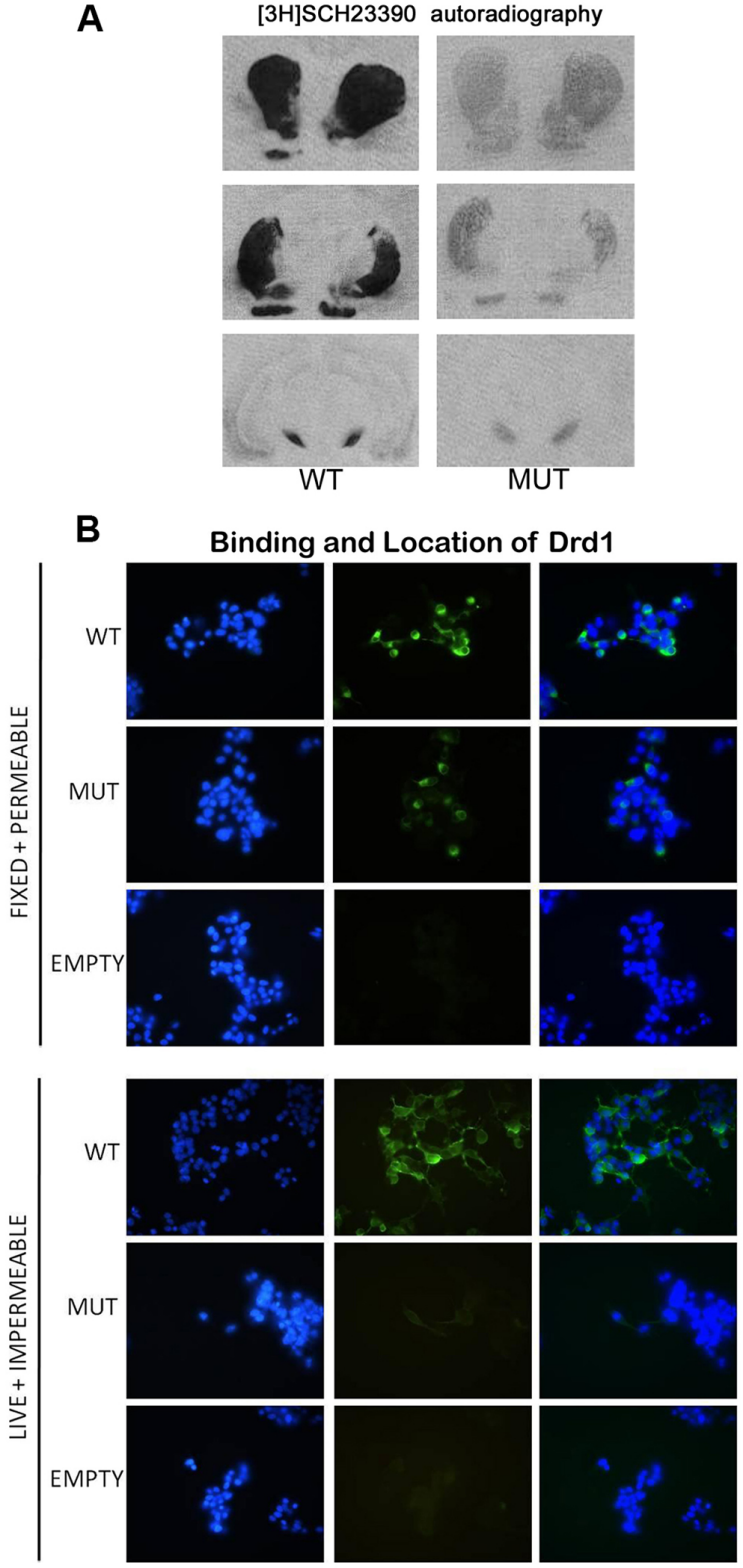
**Drd1 ligand binding and localization in wild-type and Drd1^I116S^ mutant rats.** (A) Representative [3H]SCH23390 autoradiographs of wild-type (WT, *n*=3) and Drd1^I116S^ (MUT, *n*=3) rats. A 20–50% reduction of Drd1 binding was found in Drd1^I116S^ mutants compared to wild-type rats, most likely reflecting reduced Drd1 transmembrane insertion. This experiment was replicated up to four times. (B) Upper panel, Drd1^I116S^ intracellular expression was measured in fixed and permeabilised transfected COS7 cells expressing the wild-type or mutant Drd1 receptor. The same amount of Drd1 expression was visible in the cytoplasm for the mutant and wild-type Drd1. Lower panel, Drd1^I116S^ transmembrane expression was measured in live, impermeable transfected COS7 cells expressing the wild-type or mutant Drd1 receptor. Drd1 receptor expression was reduced in impermeable COS7 cells expressing the Drd1^I116S^ compared to wild-type Drd1 receptor expression, suggesting that the Drd1 mutation affects Drd1 transmembrane insertion. Confocal pictures represent DNA staining (DAPI; blue), anti-HA staining of the Drd1 receptor (green), or DNA+Drd1 staining (blue and green merged). This experiment was executed twice. EMPTY represents cells transfected with empty vector.

To measure whether the Drd1^I116S^ mutation would affect Drd1 ligand binding we used [^3^H]SCH23390 (an Drd1 antagonist) autoradiography, focusing on the whole brain. This method measures both transmembrane and intracellular Drd1 binding, given that cells are cut at varying positions when slicing the brain. We found that [^3^H]SCH23390 binding was reduced by ∼20% (olfactory tubercle) to ∼50% (prefrontal cortex, subcortical regions, substantia nigra) in the Drd1^I116S^ mutant compared to wild-type rats (Fig. 2A; Table S1). Thus, Drd1 binding is significantly reduced in Drd1^I116S^ mutant rats. Because *Drd1* mRNA levels were unaltered, and protein synthesis presumably is unaltered as well, it is most likely that reduced Drd1 binding reflects reduced Drd1 transmembrane insertion.

### Transmembrane insertion of the Drd1^I116S^ receptor

Because receptor instability might affect transmembrane insertion of Drd1^I116S^, we measured Drd1^I116S^ cell surface expression using an *in vitro* system. We cloned the wild-type and Drd1 mutant receptor with a hemagglutinin epitope (HA) tag into COS7 cells. By using an anti-HA antibody we could identify the cellular position of the Drd1 under cell impermeable (transmembrane Drd1 identification) and permeable (intracellular Drd1 identification) conditions. We found that mutant Drd1 receptor expression was reduced in ‘live’ and impermeable COS7 cells compared to wild-type Drd1 receptor expression (Fig. 2B). The ‘fixed’ and permeabilized cells revealed that Drd1 expression in the cytoplasm was equal for the mutant and wild-type Drd1. No Drd1 immunoreactivity was observed in cells that were transfected with an empty vector. The data imply that the Drd1 mutation affects Drd1 stability and thereby its transmembrane insertion.

### Drd1^I116S^ receptor function

To assess Drd1^I116S^ function *in vivo*, we measured the effects of the D1 antagonist SCH23390 on motor reflexes in the paw test (Ellenbroek et al., 1987), a behavioural test known to be sensitive to D1 receptor antagonists. Given that the data were not normally distributed, the Mann–Whitney *U*-test was used to assess statistical differences between treatments. In wild-type rats the Drd1 antagonist SCH23390 significantly increased the forelimb reaction time (FRT; Z=−2.742, *P*<0.05) and hindlimb reaction time (HRT; Z=−2.872, *P*<0.01; Fig. S2). In addition, in Drd1^I116S^ mutant rats the FRT was significantly increased (Z=−2.605, <0.05). However, no effect was found in the HRT (Z=−1.125, not significant). Consequently, the SCH22390-induced increase in the HRT of Drd1^I116S^ rats was significantly lower than in wild-type rats (Z=−2.036, *P*<0.05). This implies that the Drd1 is less functional in Drd1^I116S^ mutant rats, potentially due to Drd1 instability and its transmembrane insertion.

### Behavioural characterization of the Drd1^I116S^ mutant rats

#### Basic behavioural assessments

To characterize the mutant rats at the behavioural level, we started with gross phenotyping using a modified SHIRPA protocol for rats (Rogers et al., 1997), focusing on motor, sensory and neurological functions. As presented in Table S2, no motor, sensory and neurological changes were found in the Drd1^I116S^ mutant rats. However, mean bodyweight was reduced in Drd1^I116S^ mutant rats compared to wild-type rats. Furthermore, upon handling, vocalization was increased in Drd1^I116S^ mutant rats. This implies changes in the social domain, which we further elaborated in the final part of this study.

Because bodyweight was significantly reduced in Drd1^I116S^ rats we measured food intake in their home cage. We found that regular eating measured over four subsequent days in the home cage (Fig. S3) was significantly decreased in Drd1^I116S^ rats [t_(1,6)_=5.947, *P*<0.05], but not when corrected for bodyweight [t_(1,6)_=0.428, not significant].

**Fig. 3.**
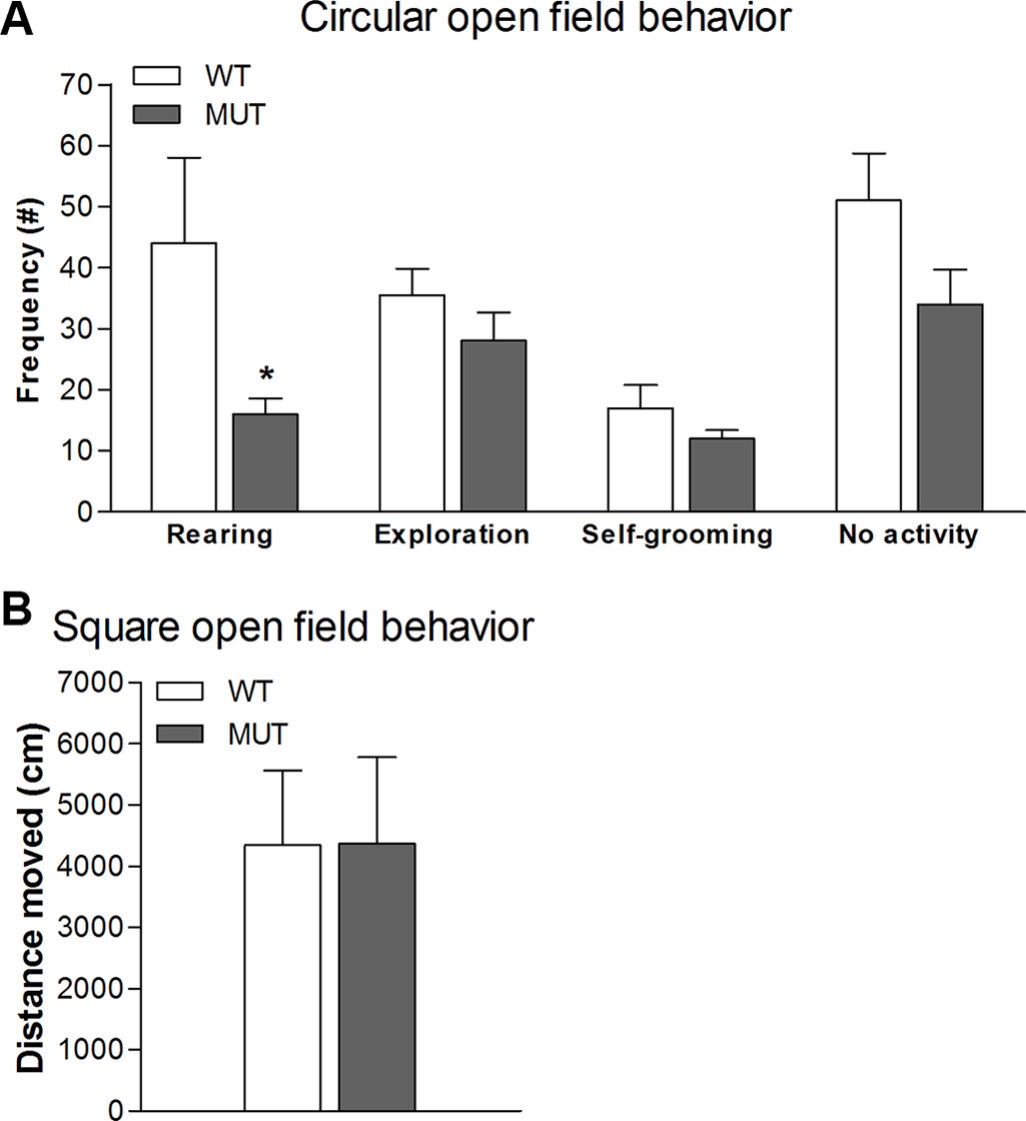
**Exploratory behaviour and self-grooming in wild-type and Drd1^I116S^ mutant rats.** Wild-type (WT) and Drd1^I116S^ mutant rats (MUT) (*n*=8) were examined for (A) frequency of rearing, horizontal exploratory behaviour and self-grooming in a circular open field. Drd1^I116S^ mutant rats displayed decreased rearing which could be an indicator of locomotor behaviour, but exploration did not differ from wild-type control animals. Self-grooming was not altered in Drd1^I116S^ mutant rats suggesting that anxiety-like behaviour is not altered. Data represent mean+s.e.m. of the frequency. (B) Distance moved in a square open field. No differences were found in distance moved indicating that the Drd1^I116S^ mutation has no effect on locomotor behaviour. Data represent mean+s.e.m. cm moved. *P<0.05 (two-tailed Student's *t*-test). The experiment was executed once.

**Fig. 4.**
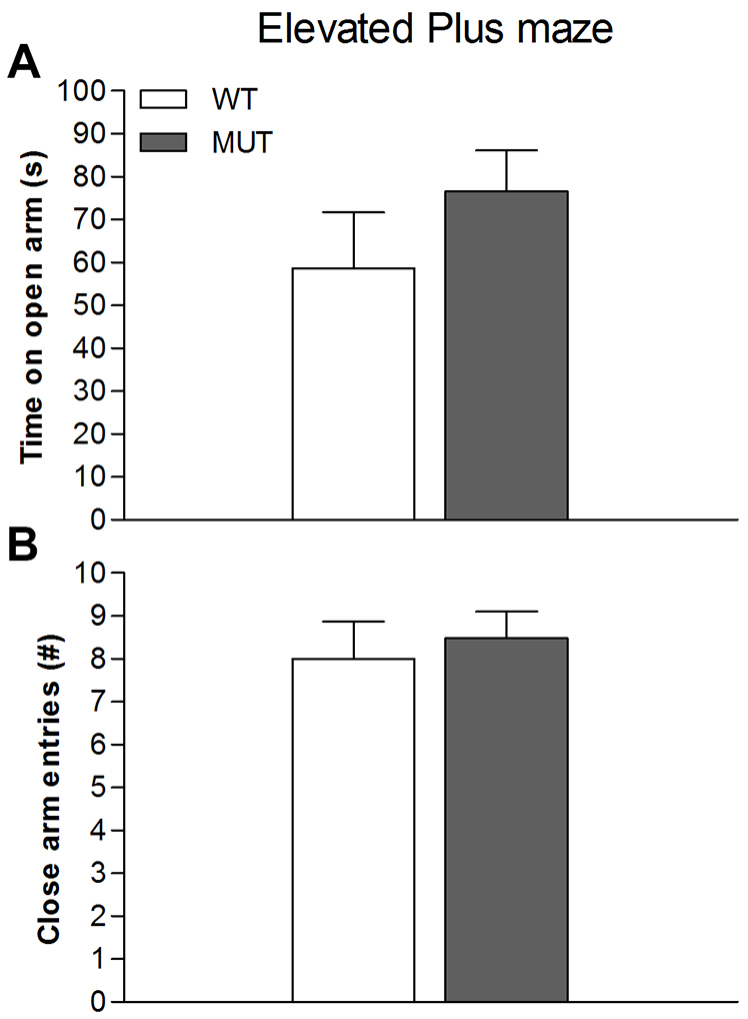
**Elevated plus-maze behaviour in wild-type and Drd1^I116S^ mutant rats.** The behaviour of wild-type (WT; *n*=20) and Drd1^I116S^ mutant rats (MUT; *n*=17) in an elevated plus maze was examined. (A) Time spent on the open arm. No differences were found between the Drd1^I116S^ mutant and wild-type rats, indicating no altered anxiety-like behaviour. Data represent mean+s.e.m. (B) Number of closed arm entries. No differences were found between the Drd1^I116S^ mutant and wild-type rats, indicating no effect of the Drd1^I116S^ mutation on general activity. Data were analysed using a two-tailed Student's *t*-test. The experiment was executed once.

Given that dopamine plays a crucial role in exploratory behaviour, we performed more sophisticated tests to assess whether behaviour in this domain would be affected by the Drd1 mutation. We observed that Drd1^I116S^ mutant rats did not differ from wild-type control animals in the frequency of horizontal exploratory behaviour bouts [t_(1,14)_=0.551, not significant] during a 30-min test in a circular open field (Fig. 3A), although rearing frequency was decreased [t_(1,14)_=2.956, *P*<0.05] in the mutant rats. Self-grooming, indicative for displacement behaviour or stereotypy, was also not affected by the Drd1 mutation [t_(1,14)_=1.179, not significant]. Additionally, in a square open field (Fig. 3B), there was no difference in distance moved between genotypes [t_(1,14)_=0.045, not significant]. Using the elevated plus maze, allowing the measurement of innate anxiety, no genotype differences were found in the time spent on the open arm [Fig. 4A; t_(1,35)_=1.079, not significant]. General activity level, as measured by the number of closed arm entries, was again normal in mutant rats [Fig. 4B; t_(1,35)_=0.159, not significant]. These tests reveal that exploratory behaviour and anxiety, with the exception of rearing, were not affected in the Drd1^I116S^ mutant rats.

### Assessment of social cognition in the Drd1^I116S^ mutant rats

#### Social interaction

**Fig. 5.**
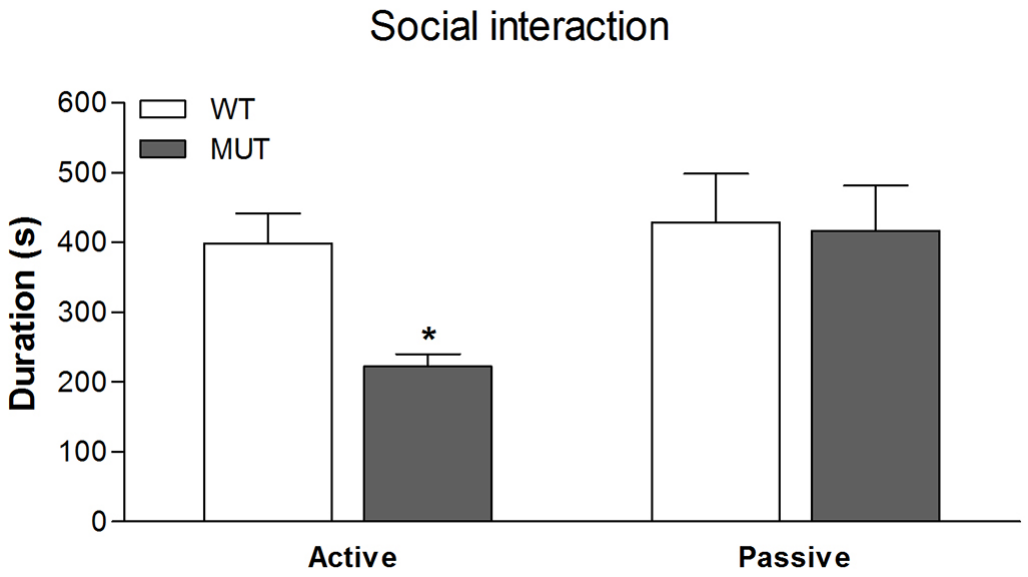
**Social interaction in wild-type and Drd1^I116S^ mutant rats.** The social interaction of wild-type (WT) and Drd1^I116S^ mutant (MUT) rats (*n*=6 pairs) was examined. A significant reduction in active social behaviour in Drd1^I116S^ mutant rats was found compared to wild-type rats, indicating a social deficit in Drd1^I116S^ mutant rats. No differences were found in passive social interaction (rats within 5 cm of each other but showing no interaction). Data are expressed as mean+s.e.m. duration of active or passive social behaviour. *P<0.005 (mixed multivariate analysis). The experiment was executed once.

First, we tested the time spent on social interaction among pairs of unfamiliar weight-matched wild-type and Drd1^I116S^ mutant rats. Compared to wild-type rats, the Drd1 mutant rats showed a profound reduction in social behaviour. This is illustrated in Fig. 5. Statistical analysis (multivariate ANOVA) showed a significant effect of genotype on active social behaviour [where one rat actively investigates (sniffing, grooming etc.) the other rat] (Fig. 5; *F*_(1,10)_=14.3; *P*<0.005), with the Drd1^I116S^ mutant rats showing consistently reduced active social interaction. No significant genotype difference was found for the duration of passive social behaviour (rats are in close proximity but do not actively interact) (Fig. 5).

#### Social approach and avoidance

**Fig. 6.**
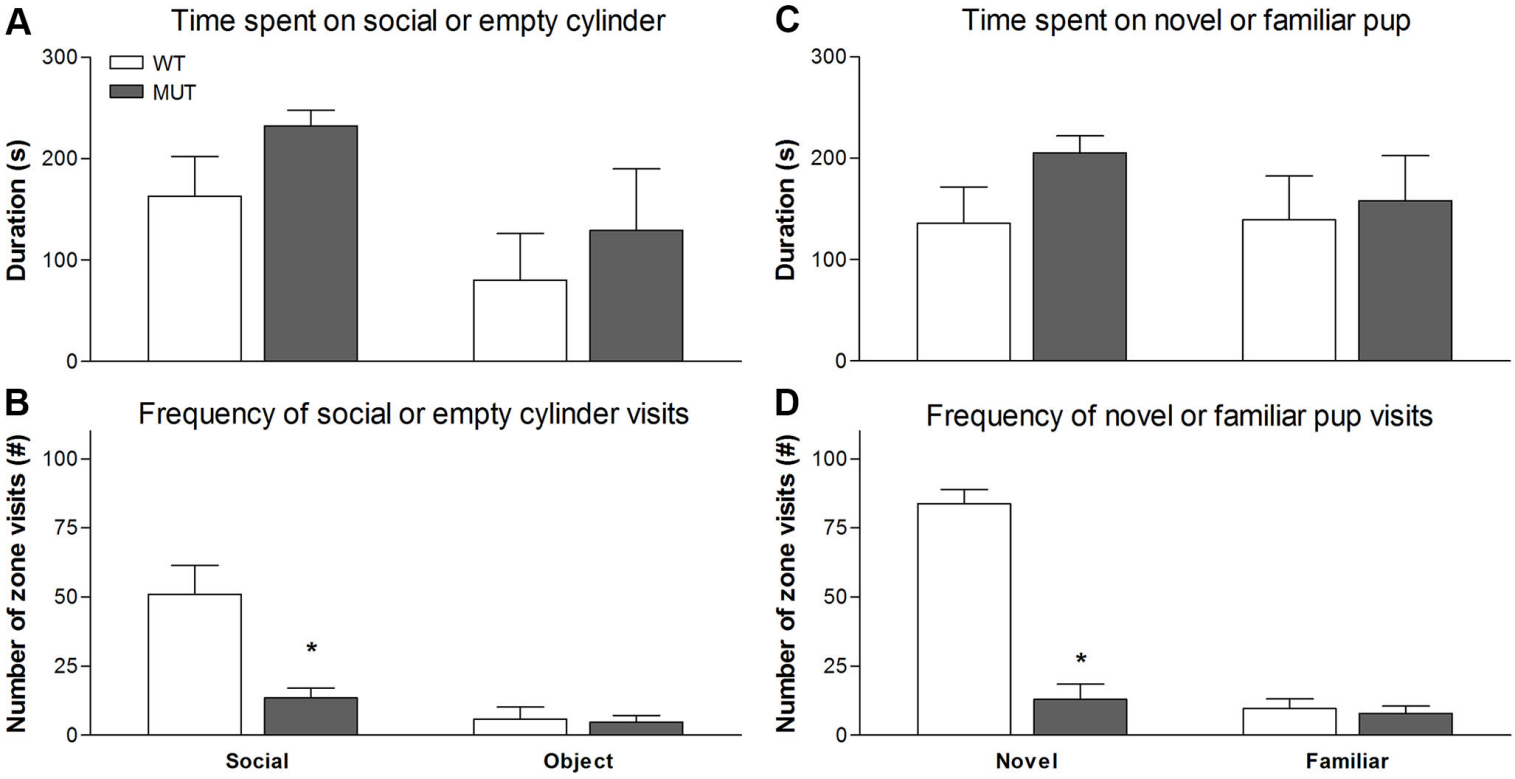
**Social approach and avoidance behaviour in wild-type and Drd1^I116S^ mutant rats.** The social approach and avoidance behaviour in wild-type (WT, *N*=7) and Drd1^I116S^ mutant (MUT, *N*=10) rats was examined. (A) Time spent on the cup with pup (social) or on the empty cup (object) in phase II. No differences were found between genotypes. Data are expressed as the mean (+s.e.m.) duration of time spent around the two cups. (B) Frequency of zone visits to the cup with pup (social) or to the empty cup (object) in phase II. The Drd1^I116S^ mutant rats had significantly fewer visits to the cup with the pup. Data are expressed as mean (+s.e.m.) number of zone visits to the two cups. These data indicate that Drd1^I116S^ mutant rats have reduced sociability in phase II. (C) Time spent on the cup with a novel pup or on the cup with a familiar pup in phase III. No differences were found between genotypes. Data are expressed as mean (+s.e.m.) duration of time spent around the two cups. (D) Frequency of zone visits to the cup with a novel pup or to the cup with a familiar pup in phase III. Drd1^I116S^ mutant rats significantly had fewer visits to the cup with the novel pup compared to the wild-type rats. Data are expressed as mean (+s.e.m.) number of zone visits to the two cups. These data suggest that Drd1^I116S^ mutant rats have a decreased interest in social novelty in phase III. *P<0.05 (mixed model ANOVA with repeated measures). The experiment was executed once.

To assess social approach and avoidance, rats were tested in a T-maze with cups containing no pup, a novel pup or a familiar pup. First (phase 1), rats were habituated to the T-maze. Next (phase 2) rats were allowed to explore the maze and encountered a pup covered by a cup on one arm, and an empty cup on the other arm. After a pause, the rats were again allowed to explore the T-maze (phase 3), now encountering a novel pup beneath the previously empty cup. The preference of the experimental animals for the T-maze arms was measured. During habituation, we found no differences between the genotypes in the total time spent on each of the two arms and total distance travelled (data not shown). The results for the second and third phase are shown in Fig. 6. Given that the data were normally distributed, a mixed model ANOVA with genotype as between and zone as within subject design was used. The data show that in phase 2, although there was a significant effect of zone [in proximity of the cup; *F*_(1,32)_=9.7, *P*<0.01], there was no significant genotype effect or interaction (*P*>0.9) for the time spent in each arm (Fig. 6A). However, with respect to zone entries, there was a significant zone [Fig. 6B; *F*_(1,32)_=10.8, *P*<0.005] and genotype effect [*F*_(1,32)_=5.5, *P*<0.05]. More importantly, there was a significant genotype×zone interaction [*F*_(1,32)_=4.9, *P*<0.05]. Inspection of Fig. 6 shows that Drd1^I116S^ mutant rats had significantly less visits to the zone with the pup. In phase 3, there was a similar difference between the time spent in the zone and zone visits. Thus, whereas no significant main effect of zone or genotype or interaction was found with respect to time spent in the two zones (Fig. 6C; all *P*>0.7), with respect to zone visits, a significant main effect of zone (*F*_(1,32)_=6.9, *P*<0.05) and genotype (*F*_(1,32)_=5.8, *P*<0.05) was seen, as well as a significant zone×genotype interaction (Fig. 6D; *F*_(1,32)_=5.2, *P*<0.05). Inspection of Fig. 6 indicated that this significant interaction was due to significant preference for the pup in WT, but not Drd1^I116S^ mutant rats. Summarizing, the data show that the Drd1^I116S^ mutant rats had a significantly reduced sociability in phase 2 and decreased interest in social novelty in phase 3.

#### Scent marking

**Fig. 7.**
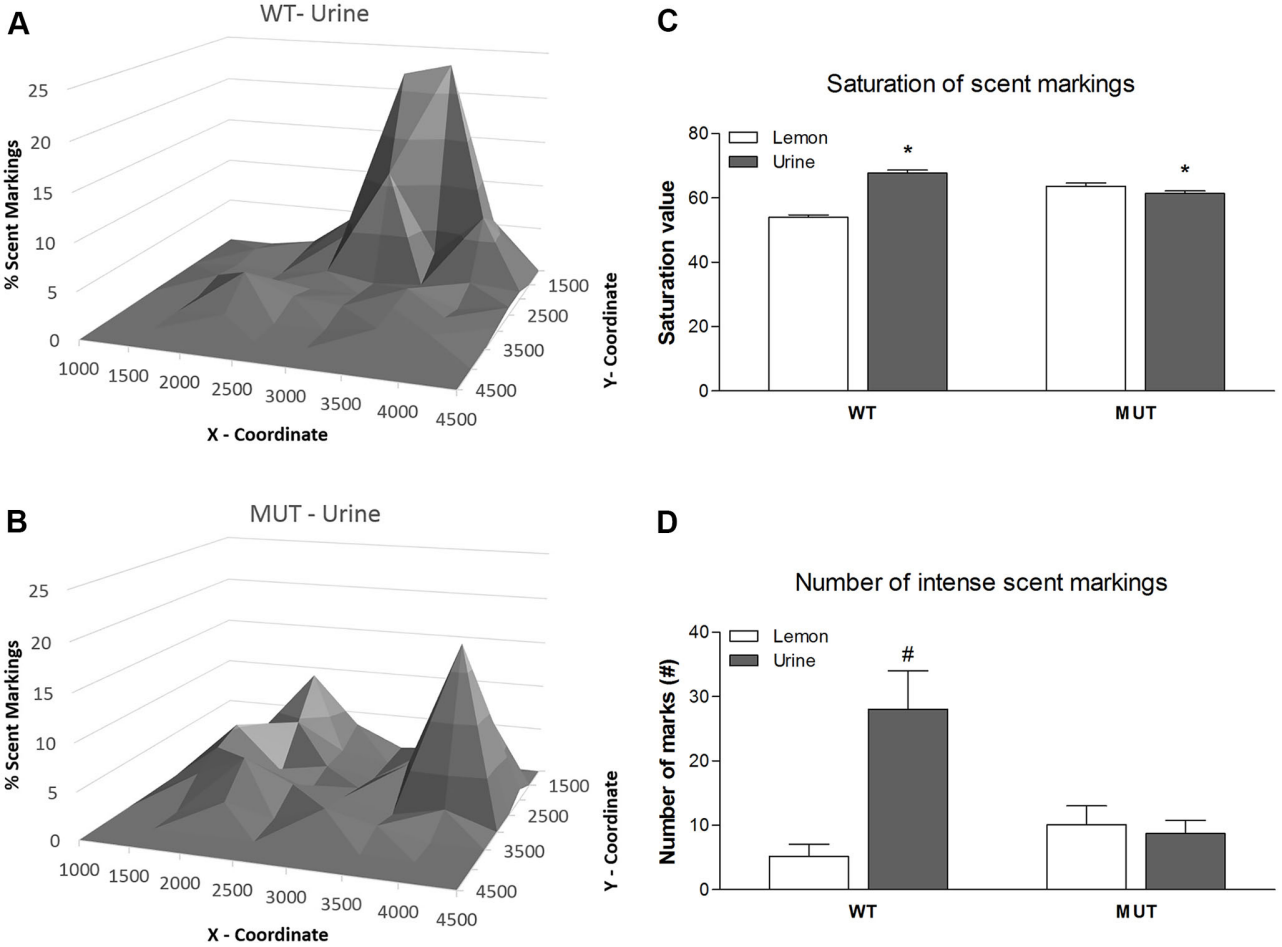
**Scent marking in wild-type and Drd1^I116S^ mutant rats.** The scent marking behaviour in wild-type (WT, *n*=14) and Drd1^I116S^ mutant (MUT, *n*=22) rats was examined. (A) Distribution of scent markings around the female urine sample by WT rats. (B) Distribution of scent markings around the female urine sample by Drd1^I116S^ mutant (MUT) rats. Wild-type control rats showed a much more restricted pattern in scent marking around the social stimulus compared with the Drd1^I116S^ mutant rats. Note, the female urine sample is placed in the centre (around coordinates 2500, 2500). (C) Mean saturation (+s.e.m.) of the scent markings around the female and the lemon scent of WT and Drd1^I116S^ mutant (MUT) rats. The wild-type rats showed a more clustered pattern around the social stimulus as compared to the non-social stimulus compared to the mutant rats. (D) Number of intense (mean+s.e.m.) scent markings around the female and the lemon scent of wild-type and Drd1^I116S^ mutant rats. Wild-type rats tended to produce much more intense markings around the social than around the non-social stimulus, whereas the reverse was true for the Drd1^I116S^ mutant rats. Together, these data suggest that Drd1^I116S^ mutants might have reduced rewarding effects of sniffing female urine. *P<0.05 urine versus lemon scent; ^#^*P*=0.08 urine versus lemon scent (a mixed model ANOVA with repeated measures). The experiment was executed once.

Using the scent marking test, odorant social communication can be measured. In this test, rats explore a lemon scent (non-social stimulus) and a female urine scent (social stimulus). The results of the scent marking experiments are illustrated in Fig. 7, showing the spatial distribution of the male urine scent markings around the social stimulus. Given that the data were normally distributed, a mixed model ANOVA was used with genotype as a between subject and scent as a within subject factor. There was a clear difference in scent marking between the genotypes, with the wild-type control rats showing a much more restricted pattern (Fig. 7A), as compared to a more diffuse pattern in the Drd1^I116S^ mutant rats (Fig. 7B) around the social stimulus. There were no clear differences in the scent marking patterns around the non-social stimulus (data not shown). Statistically, this led to a significant genotype×stimulus interaction for clustering [*F*_(1,1022)_=4.2; *P*<0.05], with the wild-type compared to the Drd1^I116S^ mutant rats showing a more clustered pattern around the social stimulus as compared to the non-social stimulus (Fig. 7C). A similar genotype×stimulus interaction was found for saturation. As illustrated in Fig. 7, wild-type rats also produced much more intense markings around the social than around the non-social stimulus, whereas the reverse was true for the Drd1^I116S^ mutant rats. However, due to large variability within genotype groups this difference failed to reach significance (Fig. 7D; *P*=0.08).

#### Ultrasonic vocalizations

**Fig. 8.**
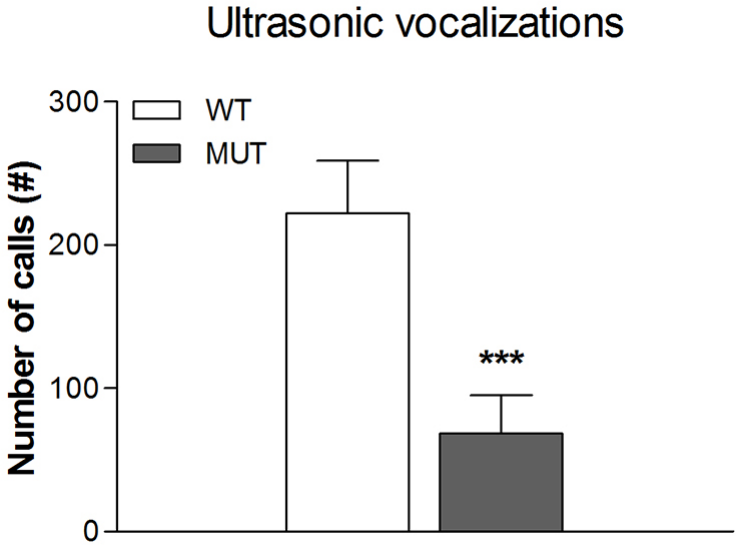
**Comparison of ultrasonic vocalizations made by wild-type and Drd1^I116S^ mutant rats.** The number of ultrasonic vocalizations made by 7-day-old wild-type (WT) and Drd1^I116S^ mutant (MUT) rats during a 5-min separation from the mother (n=8 for each genotype). Drd1^I116S^ mutant rats called significantly less frequently compared to wild-type rats, which might reflect a reward deficit. Data display (mean+s.e.m.) of ultrasonic vocalizations. ***P<0.005 (mixed multivariate analysis). The experiment was executed once.

Finally, we measured oral communication in the rats, and focussed on pups calling for their mother. When separated from their mother, young pups produce typical separation calls, in the range of 30 to 40 kHz. Although we analysed both males and females, no differences were observed and therefore results from both sexes were combined (Fig. 8). Analysis of these calls on postnatal day 14 showed that, compared to wild-type rats, Drd1^I116S^ mutant rats called significantly less [*F*_(1,16)_=11.9; *P*<0.005]. Likewise, total duration was significantly reduced in Drd1^I116S^ mutant rats [*F*_(1,14)_=8.9, *P*<0.02] whereas the average duration per call and the average frequency per call (in kHz) was not different between the different genotypes (data not shown).

## DISCUSSION

Here, we characterized a novel rat model with a genetic mutation in the dopamine D1 receptor. Although *Drd1* mRNA levels were not affected, Drd1 binding was reduced in *ex vivo* brain material of the Drd1 mutant rats. This likely is due to reduced cell membrane insertion of Drd1, as shown *in vitro* in COS7 cells overexpressing mutant Drd1. The paw test further revealed that the Drd1 in Drd1^I116S^ mutant rats was less functional compared to wild-type rats. Drd1 mutant rats did not display gross anatomical changes, although their bodyweight was significantly reduced. No differences were observed in general motor, sensory and neurological functions. Likewise exploratory behaviour and anxiety-like behaviour were not affected by the Drd1^I116S^ mutation, except for rearing. The adapted SHIRPA test revealed an increase in vocalization upon handling of the rats, hinting towards a role of the Drd1 in the social domain. In this line, several aspects of social cognition were significantly reduced in the Drd1^I116S^ mutant rats.

Computational modelling and analysis of the Drd1^I116S^ mutation suggested crucial alterations in the mutant rat model. Owing to the Drd1^I116S^ mutation, the cytoplasmic ends of helix III and helix IV cannot approach each other as closely as in the wild-type receptor. Therefore, this part of the receptor might be opened up to a larger extent, making it more accessible to G proteins. The Drd1^I116S^ mutation is also located closely to the DRY motif, which is important for interaction with intracellular G proteins, and mutations in this motif often lead to constitutive activity (Fanelli et al., 2009). Unfortunately, we have been unable to collect evidence (e.g. Drd1 agonist-induced cAMP production) for constitutive activity of the receptor. A potential reason is that, due to the mutation, the receptor quickly disassembles upon Drd1 agonist binding. These changes are also predicted to lead to lower maximum attainable activity due to decreased transmembrane insertion of the receptor (Ringkananont et al., 2006). To confirm this speculation Drd1 binding was measured in an *ex vivo* system by [^3^H]SCH23390 autoradiography in the whole brain. We found that Drd1 binding was reduced by 20–50% in Drd1^I116S^ mutant rats. However, autoradiography does not discriminate between transmembrane and intracellular Drd1 binding. To resolve this, we conducted an *in vitro* experiment in which we overexpressed wild-type and mutant Drd1 in COS7 cells, allowing us to assess the transmembrane insertion of the wild-type and mutant Drd1. We found that particularly transmembrane Drd1 insertion was reduced, with limited changes in intracellular Drd1 expression. This suggests that the Drd1^I116S^ mutation affects Drd1 stability and its transmembrane insertion, whereas the intracellular Drd1 expression remains unaltered. Reduced transmembrane insertion of Drd1 likely has consequences for Drd1 function. Indeed, in the paw test, we found that Drd1^I116S^ mutant rats were less responsive to the selective Drd1 receptor antagonist SCH23390. Although it is not yet fully clear how the Drd1^I116S^ mutation affects Drd1, the data suggest that the Drd1^I116S^ mutant rat model represents a model for reduced Drd1 function.

We used the adapted SHIRPA test for basic phenotyping of the rat model. This test did not reveal motor, sensory and neurological consequences of the Drd1^I116S^ mutation. We did observe a significantly reduced bodyweight in Drd1^I116S^ mutant rats. Given that Drd1 agonists stimulate the secretion of growth hormone from the pituitary (Bluet-Pajot et al., 1990), reduced bodyweight might be explained by reduced Drd1 function. Another possibility is that reduced food intake might cause reduced bodyweight. Of note, we observed that food intake in the home cage was reduced in Drd1^I116S^ mutants, but this difference disappeared when food intake was corrected for bodyweight. Most likely, the reduced food consumption reflects the lower calorific requirements of a smaller body. Given that animals were weight-matched in the social interaction experiments, it is not likely that decreased bodyweight influenced the differences in social behaviour.

Because dopamine plays a crucial role in exploratory behaviour, we assessed this type of behaviour in the rats when exposed to a circular and square open field, as well as the elevated plus maze. Although exploratory behaviour was not affected in these novel environments, Drd1^I116S^ mutant rats showed decreased rearing in the circular open field. Reduced rearing has been linked to decreased locomotor activity (Görisch and Schwarting, 2006; Thiel et al., 1999). However, we did not observe a decrease in locomotor activity in Drd1 mutant rats. In previous work, a decrease in rearing was also related to a decrease in food foraging, which might correspond to the reduced food consumption in Drd1^I116S^ mutant rats. The absence of exploratory changes in the elevated plus maze indicates that there were no genotype differences in anxiety-like behaviour. This was supported by the lack of genotype differences in self-grooming, which in novel environments can be seen as a stress-related displacement behaviour. Given that the previously reported relationship between Drd1 and self-grooming (Wachtel et al., 1992) is almost exclusively based on the effects of Drd1 agonists and antagonists, and because – without exception – all Drd1 ligands also bind to the Drd5, our results imply that Drd5 is more involved in self-grooming than Drd1. Taken together, these data indicate that general exploratory activity and anxiety levels are normal in Drd1^I116S^ mutant rats and do not confound behavioural measures in Drd1^I116S^ mutants.

Social cognition covers a wide variety of components, many of which are disturbed in schizophrenia and other psychiatric disorders. Social withdrawal, as measured in the social interaction test, is considered as a measure of the negative symptoms of schizophrenia (Wilson and Koenig, 2014). Withdrawal from social contact might derive from a lack of desire to have social contact. As social interactions are able to induce conditioned place preference (El Rawas et al., 2012), the social withdrawal might be explained by anhedonia, the inability to experience pleasure or reward. This explanation is supported by the scent marking test, in which male rats are allowed to approach and scent female urine, and mark the scents by urination. Given that sniffing female urine is highly rewarding for male rats (Malkesman et al., 2010), it is possible that scent marking around female urine marks was strongly reduced in Drd1^I116S^ rats due to an inability to experience female urine reward. Although we did not specifically investigate olfaction in the Drd1^I116S^ rats, the fact that there was no significant difference between the total number of scent markings between the genotypes suggests that olfaction per se was not affected. The reduction in scent markings around the social scent (and especially the altered pattern and reduced saturation) suggest that the effects are specifically related to a deficit in social cognition.

Most mammalian infants, including rat pups, vocalize when isolated. Given that interactions with adult females just before isolation increase vocalizations (a process termed maternal potentiation), it is thought that the vocalization reflects a marker of pup–mother social bonds (Shair, 2014). Expression of the maternal potentiation of the ultrasonic vocalization in pups is hypothesized to be related to reward processes, in part because dopamine activity plays a regulatory role. It has been demonstrated that activation of dopamine type-2 receptors in the nucleus accumbens blocks maternal potentiation without altering vocalization rate in an initial isolation (Shair, 2014). By contrast, it has been found that Drd1 agonists, but not antagonists, reduce the number of isolation-induced infant rat ultrasonic vocalizations (Dastur et al., 1999; Muller et al., 2009), with the latter study even reporting a significant increase with the D1 antagonist SCH23390. Here, we demonstrate that Drd1 dysfunction reduced the total number and duration of isolation-induced rat pup ultrasonic vocalizations. At present it is difficult to explain these differences, though it should be remembered that D1 agonists and antagonists are non-selective and also affect other receptors. Moreover, whereas in the present study ultrasonic vocalizations were measured at postnatal day 7, Muller and colleagues used 11- or 12-day-old pups. As ultrasonic vocalizations depend strongly on the age of the animals (Scattoni et al., 2009), this might (partly) underlie the differences between the studies. Regardless of this, the current findings are in line with the results from the other social paradigms and might reflect a reward deficit, hindering the formation of a bond with the mother. Potentially, this could lead to less maternal care (not measured in the present study) and feeding, and aberrant development of social behaviour.

Finally, sociability and social novelty as measured in the social approach and avoidance test were affected by the Drd1^I116S^ mutation. Whereas no genotype differences in duration of social approach and avoidance were found, the Drd1^I116S^ mutant rats showed a significant reduction in the frequency of visiting the area around the novel pup in phase 2. Likewise, in phase 3, whereas the wild-type rats visited the novel rat significantly more frequently than the familiar rat, this behaviour was not seen in the Drd1^I116S^ mutant rats. Analysis of the social approach and avoidance test usually includes both total time spent in the vicinity of the cylinders and total time sniffing the cylinder, with the latter being the more sensitive measure (Silverman et al., 2010). In this respect, it is important to realize that in our analysis (using Ethovision XT) we limited the analysis to the nose point, which is closely correlated to sniffing. Thus, Drd1^I116S^ mutant rats likely showed deficits in social sniffing. This is reminiscent of the finding of the social interaction test, where we found a significant reduction in active, but not passive, social behaviour in the Drd1^I116S^ mutant rats.

This study provides an initial characterization of a novel Drd1 mutant rat model, with clear endophenotypes in the social domain. Besides that the present findings raise new research questions to be addressed in future studies, we also like to mention some other potential limitations of our study. Most importantly, due to ENU mutagenesis, the Drd1^I116S^ mutant rats might bear additional mutations that have not been characterized. However, given that animals were outcrossed for at least five generations, the chance of additional mutations occuring is reduced to <1%. The Drd1^I116S^ mutant rats might share phenotypic similarities with Drd1-knockout mice, but comparison is complicated by variable findings in these mice. For instance, it has been reported that horizontal locomotor activity is unaltered (Drago et al., 1996), increased (Waddington et al., 2005) or decreased (Smith et al., 1998) in Drd1-knockout mice. Furthermore, it has been reported that Drd1-knockout mice display both reduced (Cromwell et al., 1998) and increased self-grooming behaviour (Clifford and Waddington, 1998), whereas we found no changes in self-grooming in Drd1^I116S^ mutant rats. Potentially, differences between species (or in genetic background) can account for the behavioural differences (Waddington et al., 2005). Indeed, studies have shown that the role of Drd1 in locomotor activity is fundamentally different between rats and mice (Thomsen et al., 2011). Moreover, our Drd1^I116S^ mutant rat has an outbred Wistar background, which might substantially alter Drd1 epistatic effects and approach human genetic heterogeneity to a larger extent. Finally, with the current expansion of genetic tools to manipulate the rat genome, including zinc finger nuclease, transcription activator-like effector nucleases (TALEN) and CRISPR/CAS9, ENU mutagenesis as a technology to generate mutant rat models might seem outdated (Flister et al., 2015; Parker et al., 2014). Although these more recent technologies, unlike ENU mutagenesis, allow targeted mutations, the advantage brought about by ENU mutagenesis is that it induces random mutations, not only premature stop codons and thereby knockout rats, but also hypothesis-free point mutations causing amino acid exchanges (Smits et al., 2006). With ENU mutagenesis, we generated the Drd1^I116S^ mutation. The weakness is that we do not yet completely understand how the mutation affects the D1 receptor. The strength is that the Drd1^I116S^ mutant rat displays a clear phenotype, namely a deficit in social cognition, without a complete absence of protein functioning. This might be much more relevant from a translational point of view, as complete knockouts in humans are rare. Such mutant models allow us to refine the understanding of protein conformation and function in endophenotypes of psychiatric disorders, and possible novel genetic routes to correct these.

In conclusion, we have characterized a novel genetic rat model for the Drd1 allowing the assessment of the role of Drd1 in the regulation of social cognition. The data suggest that reduced transmembrane insertion of Drd1 leads to a strong impairment in various components of social cognition. Given that rats have, compared to mice, a more extensive behavioural repertoire (Parker et al., 2014), particularly in the social domain, the Drd1^I116S^ mutant rat adds to our tools to advance the understanding of mechanisms underlying schizophrenia, autism, depression, addiction, bipolar disorder and other dopamine-related psychiatric disorders. Whereas we focussed on social cognition, this novel rat model likely also has unprecedented value for the assessment of the role of Drd1 in other behavioural domains, like reward processing and decision making.

## MATERIALS AND METHODS

### Animals

Drd1^I116S^ mutant rats were generated by ENU-driven target-selected mutagenesis on an outbred Wistar background. The Drd1^I116S^ mutant rats carry a missense mutation in Drd1, which resulted in an isoleucine to serine exchange (Drd1^I116S^) in helix III of the protein (Smits et al., 2006). Experimental animals [male wild-type (WT) and homozygous mutant (MUT)] were bred by in-crosses between heterozygous Drd1^I116S^ rats that were outcrossed for at least five generations. At the age of 3 weeks animals were genotyped. We used male rats for all experiments, unless specified otherwise. Rats were housed at two per cage in well-controlled rooms (temperature, 21°C±2°C, relative humidity, 60%±15%, light on between 07:00 and 19:00) with water and food available *ad libitum*, unless specified otherwise. Experiments were performed in separate groups of rats at adult age (10–26 weeks), between 09.00 and 16.00. Each group of rats was exposed to one test only. All experiments were conducted with the approval of the Animal Care Committee of the Radboud University in Nijmegen and the Victoria University of Wellington, according to the respective laws for experimental animals. All efforts were made to minimize the amount of animals and their suffering. Rats were not randomized, because groups were determined by genotype. *Ex vivo* and *in vitro* experiments were conducted in a blinded fashion by coding the materials. *In vivo* experiments could not be blinded, because wild-type and Drd1^I116S^ mutant rats can easily be discerned visually due to the lower bodyweight of the latter.

### Genotyping

Genomic DNA was isolated from ear cuts that were sampled in a 96-deep well block (2.5 ml Riplate, Ritter) and dissolved overnight at 55°C in 300 µl lysis buffer (100 mM Tris-HCl pH 8.5, 200 mM NaCl, 0.2% SDS, 5 mM EDTA and 100 µg/ml freshly added proteinase K). Tissue debris was spun down for 15 min at 15,000 ***g*** and supernatant was transferred to fresh tubes. DNA was precipitated by adding an equal volume of isopropanol, mixing and centrifugation at 15,000 ***g*** at room temperature. The supernatant was removed by gently inverting the block and the pellets were washed with 70% ethanol, and dissolved in 400 µl water. Genotyping was performed using the KASPar SNP Genotyping System (KBiosciences, Hoddesdon, UK) and gene-specific primers (two allele-specific oligonucleotides of ∼40 nt in length and one common oligonucleotide of ∼20 nt in length). Briefly, a PCR was carried out using the optimal thermocycling conditions for KTaq (94°C for 15 min; 20 cycles of 94°C for 10 s, 57°C for 20 s and 72°C for 40 s; GeneAmp9700, Applied Biosystems, Foster City, CA). The PCR contained 2 μl DNA solution, 1 μl 4× reaction mix, 15 pM reverse primer and 15 pM forward primer, 0.025 μl KTaq polymerase solution and 22 mM MgCl_2_ in a total volume of 4 μl. Samples were analysed in a PHERAstar plate reader (BMG Labtech, Offenburg, Germany) and data were analysed using Klustercaller software (KBiosciences). All genotypes were confirmed in an independent reaction.

### Computational analysis and modelling

Homology models of the inactive and active form of the Drd1 were obtained from the G-protein-coupled receptor data base (GPCRDB; Vroling et al., 2011). These models were built using the YASARA software (Krieger et al., 2002) using the protocol as described in Krieger et al. (2009) and alignments as provided in the GPCRDB. The structure with protein data bank (PDB) identifier 1gzm was used as template for the Drd1 in the inactive state, whereas the structure with PDB identifier 3sn6 was used as template for the active form.

### qPCR

#### Tissue punching, RNA preparation and cDNA synthesis

Snap-frozen brain samples from rats (eight wild-type and eight Drd1^I116S^ mutant rats) were partly defrosted and the striatum was bilaterally punched using a 1.2-mm punching needle (Paxinos and Watson, 2004). Brain tissue samples were homogenized with 1000 μl QIAzol Lysis Reagent (QIAGEN Sciences, MD) and 200 μl chloroform (Merck, Darmstadt, Germany). RNA was isolated with an RNeasy Lipid Tissue Mini Kit (Qiagen, 74804) according to the manufacturer's protocol. RNA concentration and quality was determined with a Nanodrop TM ND-1000 spectrophotometer (Thermo Fisher Scientific). The samples were kept at −80°C until the next day, when cDNA was made. cDNA was made with an iScript cDNA synthesis Kit (Bio-Rad, Hercules, CA) according to the manufacturer's protocol.

#### qPCR procedure

The quantitative PCR (qPCR) procedures have been described previously (Shan et al., 2012). In brief, qPCR was performed in a reaction volume of 20 µl, using the SYBR Green PCR kit (Promega, Madison, WI) and a mixture of sense and antisense primers (2 pmol/µl). Primers used for qPCR are shown in Table S3. Reactions were run in a GeneAmp 7300 thermocycler under the following conditions: 2 min at 50°C and 10 min at 95°C, followed by 40 cycles of 15 s at 95°C and finally 1 min at 60°C. Data were acquired and processed automatically by the Applied Biosystems Sequence Detection Software. Specificity of amplification was checked by means of melting curve analysis and electrophoresis of products on a 1.5% agarose gel. Sterile water (non-template control) and omission of reverse transcriptase (non-RT control) during cDNA synthesis served as negative controls.

Amplification efficiency was determined by running qPCRs on a dilution series of pooled cDNA from all the subjects. Resulting cycle threshold (Ct) values were plotted against the inverse log of each dilution and the slope of this curve was then used to calculate the efficiency as follows: efficiency (E)=10−(1/slope). The normalization factor was based upon the geometric mean of the following four reference genes selected by geNorm analysis (Vandesompele et al., 2002): tubulin-α (*TUBA*), tubulin-β4 (*TUBB4*), glyceraldehyde-3-phosphate dehydrogenase (*GAPDH*) and ubiquitin C (*UBC*). To minimize the variation, all qPCRs were conducted in duplicate.

### Quantitative autoradiography

Rats were killed by decapitation, their brains rapidly removed, frozen in liquid nitrogen and stored at −80°C. Coronal sections (16 µm) were cut on a cryostat microtome at −20°C, thaw-mounted onto gelatin-coated slides and stored at −20°C until use. Frozen sections were brought to room temperature at 60 min prior to the assay. The tissue sections (at least four slices per animal and two animals per genotype) were pre-incubated for 20 min at room temperature in 50 mM Tris-HCL buffer at pH 7.4, containing 120 mM NaCl, 5 mM KCl, 2 mM CaCl_2_, 1 mM MgCl_2,_ 1 mM EDTA, 10% w/v BSA, and 1 mM ascorbate. Subsequently, the sections were incubated in fresh buffer containing 1 nM [^3^H]SCH23390 (85.0 Ci/mmol, GE Healthcare, UK) and 40 nM ketanserin (blocking 5-HT_2_ receptors), in the presence or absence of unlabelled 2 µM butaclamol (nonspecific binding) for 1 h at room temperature. The slides were then rinsed in cold 50 mM Tris-HCl buffer for 15 s to remove superfluous radioligand, washed (4×5 min), rapidly dipped in cold distilled water and dried under a cold stream of air. [^3^H]SCH23390 sections together with [^3^H]Microscales™ standards (GE Healthcare, UK) were opposed to a [^3^H]hyperfilm (GE Healthcare, UK) and manually developed after 1 month. The [^3^H]hyperfilms were scanned using a 9200 typhoon scanner (GE Healthcare, UK). The areas of interest were determined using the Paxinos and Watson rat brain atlas, 6th edition (http://labs.gaidi.ca/rat-brain-atlas/). For each brain area, a fixed size square or rectangle box was placed in the area of interest across all slices. Using the typhoon scanner, the average pixel density within the boxes was measured. This was also done for the [^3^H]Microscales™ standards that were used in the standard curve. The optical densities within the brain regions of interest were converted into fmol/mg of tissue equivalent using this standard curve. Non-specific binding was subtracted from total [^3^H]SCH23390 binding.

### *In vitro* Drd1 mutant overexpression studies

Wild-type and mutant Drd1 was N-terminally fused to a hemagglutinin epitope (HA) tag, cloned into an expression vector pcDNA3.1 (Invitrogen) and expressed in COS7 cells. At 24 h after transfection, cells were either fixed with methanol or incubated in cold DHB medium (DMEM, 25 nM HEPES and 0.2% fatty acid-free BSA) on ice for 20 min followed by 1-h incubation on ice with rabbit 1:200 anti-HA antibody (ab9110, Abcam, Cambridge, UK) in DHB medium and methanol fixation. Samples were incubated for 1 h at room temperature with blocking buffer (1% BSA in PBS with 0.1% Tween 20). Cells were immediately fixed after the transfection procedure and then incubated for 1 h at room temperature with 1:200 rabbit anti-HA antibody (Abcam) in blocking buffer. All cells were washed with PBS three times, incubated with 1:200 goat FITC-conjugated anti-rabbit-IgG antibody in blocking buffer for 1 h in the dark. After three washes with PBS, coverslips were mounted using Vectashield with DAPI (Brunschwigchemie, Amsterdam, The Netherlands) and cells were analysed using confocal microscopy. The cells were checked for mycoplasma contamination, and found to be negative.

### Basic behavioural characterization of the Drd1^I116S^ mutant rats

#### Adapted SHIRPA

The SHIRPA protocol was developed as a quick screen for general measures of health, motoric and neurological parameters in genetically modified animals. The procedure described by Rogers et al. (1997) was modified for the rat. Rats were placed in a Perspex jar on a grid for 5 min and evaluated for body position, spontaneous activity, respiration, body tremor and number of fecal boli. Immediately thereafter, rats were placed in a wooden box with walls (57×57×28 cm). The floor contained separated back line drawings of 16 squares. The following behaviours were recorded: transfer arousal, latency time to walk to the walls, locomotor activity, eye opening, gait, particularities of the body hair, pelvic elevation, tail position, approach reaction and touch escape. The rats were then lifted by the tail and assessed for the position at which struggling movements occurred, as well as trunk curl, head righting reflex and clenching with the paws to the tail. At landing on the horizontal grid, the rats were evaluated for grasping the grid bar, grip strength when dragged by the tail across the grid, body tone after finger compression on each side, pinna, corneal and whisker reflexes, as well as toe-pinch withdrawal of the hind limb after squeezing with hand-held forceps. The forepaws were then placed on a horizontal bar and the ability of the rats to hang suspended was evaluated. Furthermore parameters like skin colour, hind limb tone, abdominal tone, lacrimation, salivation and provoked biting in response to a pair of tweezers being put in their mouth were observed. The air-righting reflex, together with contact righting when placed inside a small plastic tube were evaluated followed by the postural effects. Finally, rats were placed on a horizontal grid, rotated toward a vertical position of 45° and assessed for negative geotaxis, and behaviours like freezing behaviour, irritability, aggression and vocalization were scored. For all rats, the bodyweight, nose-to-tail length and rectal body temperature was measured.

#### Food consumption in the home cage

Rats were housed in genotype-matched pairs and the wirebar lids containing standard food pellets were weighed during four consecutive days. In order to correct for bodyweight rats were weighed on the first day of measuring as bodyweight was not expected to change during this short period.

#### Paw test

The paw test was developed to assess dopaminergic functioning and consists of a Perspex platform (30×30×20 cm) containing four holes, two hindlimbs holes (diameter: 5 cm), two forelimbs holes (diameter of 4 cm) and a slit for the tail (Ellenbroek et al., 1987). The distance between the left and right forelimb and hindlimb holes was 15 mm, and the distance between forelimb and hindlimb holes was 55 cm. At 30 min before testing the rats were treated with SCH23390 (1 mg/kg bodyweight, intraperitoneal injection). The rats were placed on the platform by inserting the hindlimbs and subsequently the forelimbs in the holes. Hind limb retraction time (HRT) and forelimb retraction time (FRT) were measured, with a minimum of 1 s and a maximum of 30 s. The test was repeated at 40 and 50 min after injection. The three measurements were averaged.

#### Novelty-induced exploratory behaviour and self-grooming

Rats were placed in a transparent circular open field (diameter 20 cm) for 30 min, and behaviour was videotaped. Exploratory behaviour, rearing, the duration and frequency of total grooming, and face and body grooming were analysed using Observer (Version 3.1, Noldus Information Technology, Wageningen, The Netherlands). Additionally, another group of animals was tested in a square open field measuring 50×50×50 cm for 30 min. Locomotor activity (distance moved, cm) was monitored using EthoVision (Version 3.1, Noldus Information Technology).

#### Elevated plus-maze test

The apparatus was made of grey PVC and elevated 75 cm above the floor. The four arms (50×10 cm^2^) formed a cross with the central platform. A wall (height: 30 cm) of non-transparent material enclosed two arms, located opposite to each other. Each rat was placed on the central platform facing one of the enclosed arms and allowed to freely explore the maze for 5 min. Behaviour was scored manually using Observer 5.0 (Noldus Information Technology, Wageningen, The Netherlands). The time spent on the open arm of the maze was calculated as a measure of anxiety, whereas the total number of closed arm entries was considered as a measure of general activity.

### Assessment of social behaviour in the Drd1^I116S^ mutant rats

#### Social interaction

Adult rats were isolated for 3 days prior to the experiment to enhance the display of social behaviour. On the day of the experiment, pairs of rats (of similar age, sex and genotype) from different litters were placed in a standard translucent polypropylene box (40 cm×72 cm×22.5 cm high), under dim light conditions. The beddings of dust-free wood chips from both subjects' cages were combined and transferred to the experiment box in order to reduce the novelty of the environment. The behaviour of the rats was recorded for 30 min with a video camera mounted above the cage. Duration and frequency of the following behaviours was scored offline using Observer^®^ (Noldus Information Technology, Wageningen, The Netherlands) (blinded for genotype): (1) active social behaviour, both rats are in close proximity with each other and at least one of the rats is actively investigating (sniffing, grooming etc.) the other rat; (2) passive social behaviour, both rats are in close proximity (within ∼5 cm of each other) but neither animal shows active social investigation; (3) non-social behaviour, rats are further than 5 cm away from each other.

#### Social approach and avoidance

Social approach avoidance was measured in a T-maze (arms 50 cm long, 20 cm wide, with 25-cm-high walls) using a three-phase paradigm. In the first phase (habituation), rats were placed in the T-maze and allowed to explore the maze. After 15 min the animal was removed and the maze cleaned with 70% ethanol. Next, two small cylindrical cups (9 cm diameter) were placed upside down at the end of each of two arms. Under one cup a juvenile rat was placed, while the other cup was empty. The experimental rat was placed back and was able to explore the cups for 5 min. After this second phase, the rat was removed and a new juvenile rat was placed under the previous empty cup, while the familiar pup stayed in the previous cup. The experimental rat was placed back again and for a final 5 min was allowed to explore the T-maze. The behaviour of the experimental rats was measured using Ethovision XT v.9. This program allows for a detailed tracking of behaviour, detecting nose-point, central body-point and tail-base point separately. For this analysis, the total time the nose-point was within a zone of 5 cm of each of the cylinders was analysed. In addition, the frequency of (nose-point) zone visits was analysed.

#### Scent marking

Scent marking was measured using a protocol very similar to that developed for mice (Wohr et al., 2011). Briefly, adult male rats were familiarized with female rats (from the same genotype) by placing them together with a female for a period of 5 min between 5 and 7 days before the experiment, to allow them to experience female scent. On the day of the experiment, rats were allowed to habituate to a novel round open field (diameter 80 cm). After 15 min the experimental rat was removed and the open field cleaned. Two circular pieces of filter paper (diameter 30 cm) were placed in opposite quadrants of the open field, one impregnated with 30 μl of lemon scent (non-social stimulus) and one with 30 μl of fresh urine (social stimulus) from females in oestrus. Females that were in oestrus (as determined by a vaginal smear; Marcondes et al., 2002) were gently held between the forelimbs. This was usually sufficient to induce urine flow. This urine was collected in Eppendorf tubes and used within 1 h after collection.

After both filter papers were impregnated with the smells, the experimental male rat was placed back and allowed to explore the open field for an additional 5 min. After this period the animal was removed, the filter papers sprayed with ninhydrin spray (which dyes amino acids) and dried overnight. The male urine scent markings left on the filter paper sheets were analysed by an open source software (openCFU) designed to count small circular objects (for the details of the program, see Geissmann, 2013). To differentiate between normal micturition and scent markings (which are much smaller), a maximum filter size of 10 pixels was applied to the image processing. The variables analysed were the number of male urine markings surrounding the target scent, the size of each marking (radius), saturation (intensity of each marking) and clustering (the number of markings in close proximity to another marking).

#### Ultrasonic vocalizations in young rats

Ultrasonic vocalizations were recorded in 7-day-old male pups. Rat pups from different litters were taken from their mother and placed in a small circular container (diameter 9 cm) with fresh bedding material on the floor. Ultrasonic vocalizations were recorded for a period of 5 min from individual pups using Ultravox XT^®^. The same program was used to count all calls within the 30–50 kHz range. Both the total number of calls and total duration were recorded.

### Statistical analyses

Data were checked for normality and homogeneity and analysed using Student's *t*-tests (home-cage food consumption, novelty-induced exploratory behaviour, self-grooming and elevated plus maze), mixed multivariate tests (social interaction and ultrasonic vocalizations), mixed model ANOVA with repeated measures (social approach and avoidance, scent marking), a χ-squared test (adapted SHIRPA) or a Mann–Whitney *U*-test (qPCR and paw test). All data were analysed using SPSS 16.0 software (LEAD technologies, Chicago, IL). No a priori power analysis was conducted; because of the novelty of the rat model, a priori data for a power analysis were not available. The level of significance was set at *P*<0.05. Data are expressed as mean±s.e.m.
